# Crystallization Kinetics of Oleogels Prepared with Essential Oils from Thirteen Spices

**DOI:** 10.3390/foods14030542

**Published:** 2025-02-06

**Authors:** Wei Zhou, Lin Yu, Zihao Wei

**Affiliations:** 1 College of Food Science, Henan Institute of Science and Technology; Xinxiang 453003, China; zhouwei1981@hist.edu.cn (W.Z.); yl126789@163.com (L.Y.); 2 College of Food Science and Engineering, Ocean Univeristy of China, Qingdao 266404, China

**Keywords:** thirteen spices essential oils, glycerol monostearate, oleogels, crystallization kinetics

## Abstract

In this study, corn oil and essential oils from thirteen spices were used as the oil phase, with glyceryl monostearate (GMS) serving as the gelling agent to prepare the oleogels. The effects of varying the concentrations of the gel additives (2%, 4%, 6%, and 8%) on the texture, oil retention, and rheological properties of the oleogels were investigated using differential scanning calorimetry (DSC) and X-ray diffraction (XRD). The results showed that GMS concentration markedly influenced the structure and properties of the gel. Positive correlations were observed between GMS concentration and the results of texture analysis, oil binding capacity, and microscopic morphology of the oleogels. Analyses via DSC and XRD demonstrated that gel formation was attributable to the crystalline network induced by GMS. Rheological assessments revealed that the oleogels exhibited pseudoplastic behavior and commendable thermal sensitivity.

## 1. Introduction

Oil gelling technology represents an innovative approach in food processing, where a small amount of gelling agent is added to vegetable oil to form a tough, viscoelastic, semi-solid lipid matrix. This unique structure effectively immobilizes liquid fats and oils by creating a three-dimensional network that restricts molecular mobility [1,2]. Compared to traditional liquid oils, solid fats are widely used in the food industry to enhance texture, mouthfeel, and flavor, as well as to provide customizable rheological and thermal properties [3]. However, with growing awareness of the adverse effects of excessive saturated and trans fat consumption, such as increased risks of cardiovascular diseases, type 2 diabetes, and metabolic syndrome [4,5], there is a pressing need to develop oil gelling technologies that can increase unsaturated fat content while reducing harmful fat intake. This has become a critical focus in food science research [6].

Since its inception in 1992, oil gelling technology has undergone significant advancements. Early studies demonstrated the feasibility of forming oil-based gels by adding ethyl cellulose to the oil phase and heating it to 140 °C, laying the groundwork for subsequent research [7]. The field has since expanded to include edible oil gels, broadening their application and deepening our understanding of their structure and health implications. The publication of the seminal book *Edible Oleogels: Structure and Health Implications* further catalyzed research in this area, particularly in reducing saturated fat content in processed foods like Frankfurt sausages [8]. Studies on various vegetable oils, such as sunflower and hazelnut oils, have demonstrated the versatility of oil gelling technology in food preparation, including ice cream, spreads, biscuits, chocolate, and muffins [9,10,11,12]. These achievements underscore the importance of oil-gelling technology in food formulation and processing, while also paving the way for future innovations.

Among the various gelling agents, glyceryl monostearate (GMS)—a low-molecular-weight, non-ionic surfactant—has garnered significant attention due to its unique properties. GMS serves as both a lipid structurant and an emulsifier, making it a cost-effective and safe option for food applications [13]. Its ability to efficiently construct lipid structures at low concentrations is particularly advantageous for the food industry [14]. As a single-component oil gel factor, GMS can self-assemble in both aqueous and hydrophobic media, offering opportunities for developing new food ingredients and improving food structures. The reverse lamellar bilayer structure formed by GMS in vegetable oils, along with its stable polymorphic β-phase, further highlights its potential in food science and technology [15]. In-depth research on GMS and its application in oil gel systems could not only advance food processing technologies but also contribute to the development of healthier and more innovative food products.

While organogel technology has shown promising advances in food applications, the incorporation of essential oils (EOs) into organogel systems presents both opportunities and challenges [16,17]. EOs are valuable ingredients in foods, pharmaceuticals, and cosmetics, offering unique aromatic properties and potential health benefits [18,19]. However, their practical application is often limited by characteristics such as strong odor, irritability, and volatility [20]. The development of EO-based organogels represents a potential solution to these challenges by providing enhanced stability and controlled release capabilities.

Among various EOs, essential oils from thirteen spices emerge as particularly interesting candidates for organogel applications. This traditional Chinese spice blend contains a complex mixture of bioactive compounds, including anethole, limonene, and various fatty acids (Supplementary Table S1). Unlike single-component EOs that have been extensively studied, the behavior of such complex spice blends in organogel systems remains largely unexplored. Previous research has demonstrated the effectiveness of various EOs in food applications—from enhancing antioxidant properties in French fries to reducing lipid oxidation in virgin olive oil and meat products [21,22,23,24]. The multi-component nature of EOs from thirteen spices not only provides distinctive aromatic properties but also offers potential functional benefits such as antioxidant and antimicrobial activities.

This research aims to optimize the preparation of oil gels using GMS as a structuring agent and EOs from thirteen spices as a functional component. Through comprehensive characterization of physicochemical properties, including texture, oil retention, rheological properties, XRD, and DSC analyses, this study seeks to bridge the gap between traditional Chinese culinary wisdom and modern food technology. The findings are expected to provide fundamental insights for the practical application of complex essential oil systems in innovative food products.

## 2. Materials and Methods

### 2.1. Materials

Corn oil was provided by Yihai Kerry Food Marketing Co., Ltd. (Shanghai, China). The fatty acid composition of the corn oil was as follows: linoleic acid (ω-6, 50–52%), α-linolenic acid (ω-3, ~1%), oleic acid (27–30%), palmitic acid (11–13%), and stearic acid (~2%). The oil contained vitamin E (100–120 mg/100 g) and had an acid value of ≤0.3 mg KOH/g and a peroxide value of ≤10 mmol/kg.

Essential oils from thirteen spices (EOs) were supplied by Zhengzhou Semillon Food and Fragrance Co., Ltd. (Zhengzhou, China). The composition and content of the essential oils were analyzed by GC-MS, and the testing services were provided by the Science Guide Testing Platform of Hangzhou Yanqu Information Technology Co., Ltd. (Hangzhou, China). Detailed data are presented in Supplementary Table S1.

Glyceryl monostearate (GMS) with a purity of 95% (CAS No. 31566-31-1, chemical formula C_21_H_42_O_4_) was obtained from Shanghai McLin Biochemical Technology Co., Ltd. (Shanghai, China).

### 2.2. Preparation of Oleogels

The preparation method of oleogels was adapted from the studies by Jiang et al. (2019) with slight modifications [25]. GMS at concentrations of 1, 2, 4, 6, and 8% was added to corn oil and heated in a constant-temperature magnetic stirrer at 90 °C until completely dissolved. After cooling to 25 °C, 15 μL/mL of EOs from thirteen spices was added. The prepared samples were stored at 4 °C and 25 °C for 24 h to form oleogels. Keeping the GMS concentration at 8%, samples were stored for 24 h to form oleogels by adding EOs from thirteen spices at concentrations of 0, 5, 10, 15, 20, and 25 μL/mL. The prepared oleogels were inverted at room temperature; the flowing oleogels indicated a non-gel state, and the non-flowing oleogels indicated a gel state. By observing the fluidity and color of the samples, the limits of the amounts of EOs from thirteen spices and the gelling concentration of the glycerol esters were determined.

### 2.3. Measurement of Oil Binding Capacity

The oil binding capacity (OBC) was determined according to the method of Liu et al. (2020) [26]. First, the empty centrifuge tube was weighed (*a*). Approximately 2 g of the oleogel sample was then placed in the centrifuge tube, and the mass was recorded (*b*). The sample was centrifuged at 9000 rpm for 15 min. After centrifugation, the tube was inverted on a filter paper for 15 min to completely drain any unbound oil, and the total mass was measured (*c*). The OBC (H) was calculated using the following formula:

(1) $$H=\frac{c-a}{b-a}100\%.$$

### 2.4. Determination of Textural Properties

The mechanical properties were analyzed using a TA-XT plus texture analyzer (Stable Micro Systems Ltd., Surrey, UK). Approximately 10 g of the sample was used for each test. Textural properties were assessed using a p/0.5 R probe (0.5 inches in diameter, 40 mm) in the compression mode. The test was conducted with two compressions at a descent rate of 1 mm/s (pre-test rate of 1 mm/s, mid-test rate of 1 mm/s, and post-test rate of 1 mm/s), a compression ratio of 75%, and a triggering force of 5 g with intervals of 5 s. Hardness, as defined in the test results, was the highest peak observed during the first compression. Brittleness was defined as the second-highest peak. Viscosity was represented by the negative area under the curve of the first compression. Chewiness was calculated as the product of hardness and viscosity.

### 2.5. Determination of Thermal Behavior

The DSC measurements were performed using a differential scanning calorimeter (DSC3, Mettler Toledo, Greifensee, Switzerland). Approximately 10 mg of the sample was placed in an aluminum pan, with an empty pan serving as the control. The test procedure was as follows: initially, the sample was heated to 90 °C for 20 min to eliminate any memory of crystallization, then cooled to 5 °C at a rate of 2 °C/min and held for 10 min, and finally, the temperature was increased to 90 °C at a rate of 5 °C/min [27].

### 2.6. Rheology Test

The rheological properties of the samples were characterized using a rotational rheometer (HAAKE MARS III, Thermo Scientific, Waltham, MA, USA). Approximately 1 g of the sample was used for each test. A parallel plate fixture, P35TiL, with a diameter of 35 mm was utilized, and the gap was set at 1.000 mm. The linear viscoelastic region (G′ LVR) of the oleogels was identified through stress scanning, setting the stress range to a logarithmic change from 0.1 to 100 Pa at a constant frequency of 1 Hz. Initially, dynamic measurements were conducted where the shear rate (γ) varied from 0.1 to 100 s^−1^, while the shear stress (τ) was maintained at 1 Pa. Subsequently, the gel temperature was determined using a variable temperature program, with the measurement frequency fixed at 1 Hz and the shear stress at 1 Pa. The samples were heated to 90 °C for 10 min to completely eliminate any crystallization memory and then cooled to 0 °C at a cooling rate of 3 °C/min.

### 2.7. X-Ray Diffraction

XRD was employed to analyze crystallinity using an X-ray diffractometer (D8 Advance, Bruker, Germany). Approximately 20 mg of the sample was prepared and placed in the testing chamber. The testing conditions were established as follows: utilizing a Cu radiation source (wavelength λ = 1.54056 Å), with a working voltage of 40 kV, a working current of 40 mA, and a scanning range of the θ angle from 10.0° to 50.0°. The scanning step size was set at 0.05° with a scanning rate of 17.7 s per step. The emission and anti-reflection slit was 1.0 mm, the receiving slit was 0.1 mm, and the testing temperature was maintained at 20 °C. The scanning spectra were analyzed using Jade 5.0 software.

### 2.8. Statistical Analysis of Data

Measurements for each group were repeated three times, and the results are presented as mean ± standard deviation (SD). Data processing and graphing were conducted using GraphPad PRISM9. Data analysis was performed using one-way analysis of variance (ANOVA) with SPSS 26.0 software. Duncan’s test was used for comparison purposes, with the significance level set at *p* < 0.05.

## 3. Results and Discussion

### 3.1. Analysis of Gelling Properties

**Figure 1:**
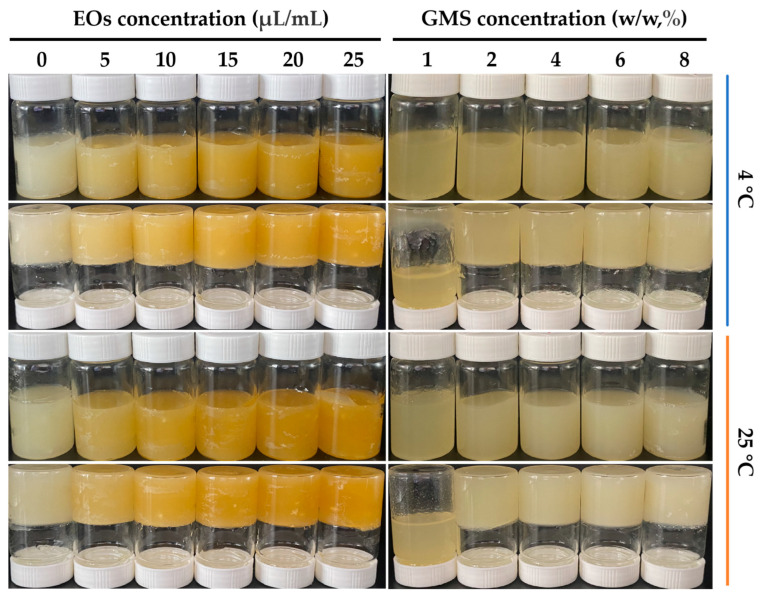
Effect of EOs concentration on the structuring of 8% GMS-loaded oil at different cooling temperatures. EOs refer to essential oils from thirteen spices; GMS refers to glyceryl monostearate.

The curing of oleogels was observed using the inverted bottle method, and the effects of GMS concentration, the concentration of EOs from thirteen spices, and storage temperature were examined. The GMS concentrations tested were 2, 4, 6, and 8% *w*/*w*, and the concentration of the EOs from thirteen spices was set at 0, 5, 10, 15, 20, and 25 μL/mL. Storage temperatures were maintained at either 4 °C or 25 °C, as shown in Figure 1. At both 4 °C and 25 °C, when the GMS concentration was at or above 2%, the system remained in a non-flowing state for 24 h, indicating the formation of oleogels. This behavior is attributed to the structure of GMS itself, which is an esterification product of glycerol and stearic acid, comprising varying amounts of monoglycerides, diglycerides, and triglycerides. Consequently, liquid oil is immobilized, leading to the formation of more crystals and the strengthening of the crystallization network [28].

Oleogels containing the EOs from thirteen spices appeared yellow, whereas those without these oils were lighter and whiter in color. As indicated in Table 1, the photometric (L*) values of the samples significantly increased with increasing concentrations of the EOs from thirteen spices (*p* < 0.05). The increase in L* value may be attributed to the presence of these EOs. The a* and b* values, which represent green and red colors, respectively, also increased with increasing concentrations of the EOs (*p* < 0.05). Taking into account the appearance and aroma of the oleogels, this experiment utilized a concentration of 15 μL/mL for the Eos from thirteen spices.

### 3.2. Oil Binding Capacity

**Figure 2:**
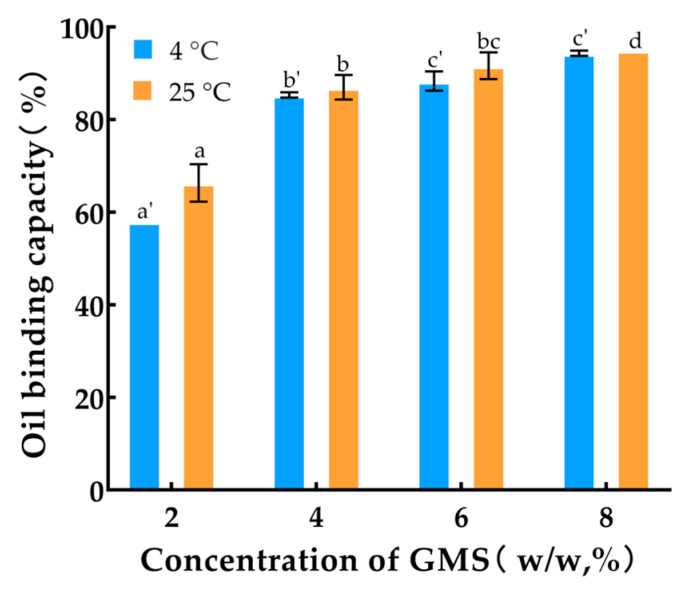
Oil binding capacity of 15 μL/mL EOs-containing oleogels with various concentrations of GMS. The error bars indicate the standard deviation of the mean for n = 3 samples, and lowercase letters represent significant differences between samples in each column (*p* < 0.05).

The OBC of the oleogels prepared at various concentrations and cooling temperatures is shown in Figure 2. The results indicate an increase in OBC as the GMS mass fraction increased. A higher quantity of gelling agent led to a denser and stronger gel network, which in turn increased the content of bound liquid oil. When the glyceride mass fraction was at or above 4%, the oil retention rate of the oleogels remained relatively stable across different storage temperatures, all exceeding 80%. This finding suggests that the GMS-type oleogels exhibited excellent resistance to the migration of oil esters.

At an 8% addition of gelling agent, the oil retention capacity of oleogels stored at 4 °C and 25 °C was comparable, indicating that the crystalline state of GMS had reached saturation. The increase in OBC could be attributed to a minor substitution in oleogels that enhanced the nucleation and crystallization processes, with a considerable amount of corn oil being captured by the oleogels within the network. Consequently, the capacity of GMS to bind to liquid oil also peaked.

### 3.3. Texture Analysis

The textural characteristics of the prepared oleogel samples under different GMS concentrations and storage temperatures were analyzed using a texture analyzer, as shown in Table 2. The results demonstrated that the preparation conditions significantly influenced the textural properties of the oleogels. The GMS concentration was positively correlated with the hardness, brittleness, viscosity, and chewiness of the oleogels (*p* < 0.05). At the same storage temperature, the textural properties of the oleogels improved with increasing GMS concentration.

Higher texture profile analysis (TPA) values were observed with an increase in the proportion of GMS in the coagulant combination. These higher TPA values corresponded with increased OBC and melting temperature of the oleogel samples, which are key indicators of ideal oleogels. This suggests that a smaller amount of oleogel can achieve the desired physical properties without significantly affecting the sensory characteristics of the food product. Additionally, intermolecular hydrogen bonds within the three-dimensional network structure of the oleogels may have contributed to the increased hardness.

The experimental results also showed that high gelator concentrations and low storage temperatures increased the number and strength of crystals in oleogels. By adjusting the GMS concentration and storage temperature, the textural properties of the oleogels can be tailored to meet various production requirements.

### 3.4. Analysis of Thermodynamic Properties

**Figure 3:**
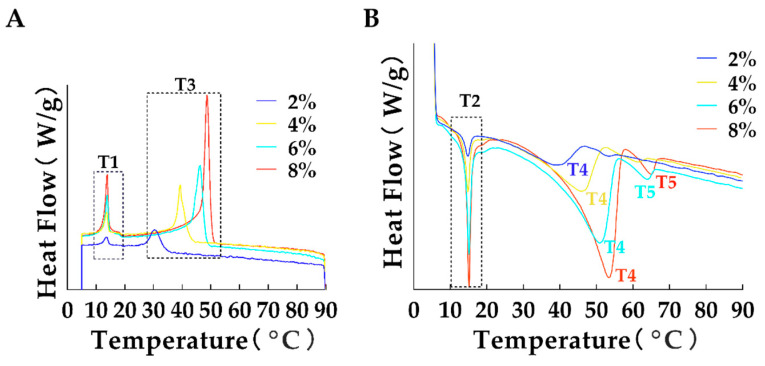
Thermal stability of 15 μL/mL EOs-containing oleogels with various concentrations of GMS. (**A**) Crystallization curve of oleogels; (**B**) melting curve of oleogels. T1, T2: lower peak temperatures; T3, T4, T5: higher peak temperatures.

The functional properties and stability of oleogels are closely related to their thermal characteristics [29]. This study evaluated the effects of the crystallization and melting behaviors of GMS on its binding efficiency. The thermodynamic properties of oleogels with varying GMS content were analyzed using DSC, with the results shown in Figure 3 and Table 3. Figure 3 presents the non-isothermal crystallization and melting curves of oleogels with different GMS concentrations. The differences in solid solution formation can be attributed to solute-solute interactions among the various components of the oleogels [30]. Both the melting and crystallization peak temperatures increased with higher GMS content, significantly enhancing their thermodynamic stability of the oleogels (*p* < 0.05). The crystallization peaks exhibited a bimodal pattern, and the melting peaks varied accordingly. Specifically, two melting peaks were observed at 2% and 4% GMS content, while three melting peaks appeared at 6% and 8%, which can be attributed to the crystallization behavior of GMS within the three-dimensional structure and its high cohesive energy.

The observed thermal characteristics align with previous research findings on similar systems [31,32]. The progressive increase in both crystallization and melting temperatures with higher GMS concentrations suggests improved structural stability of the oleogel system. These thermal properties were comparable to those of commercial shortening, indicating the potential practical applications of these oleogels.

### 3.5. Analysis of Rheological Properties

Rheological analysis, which provides valuable insights into the microstructural behavior of oleogels, evaluates the storage modulus (G′) and loss modulus (G″) under both stable and dynamic conditions [25]. This method is instrumental in understanding the deformation behavior within the linear viscoelastic region (LVR). To characterize the rheological behavior of oleogels, parameters such as the storage modulus (G′), loss modulus (G″), and apparent viscosity were employed.

**Figure 4:**
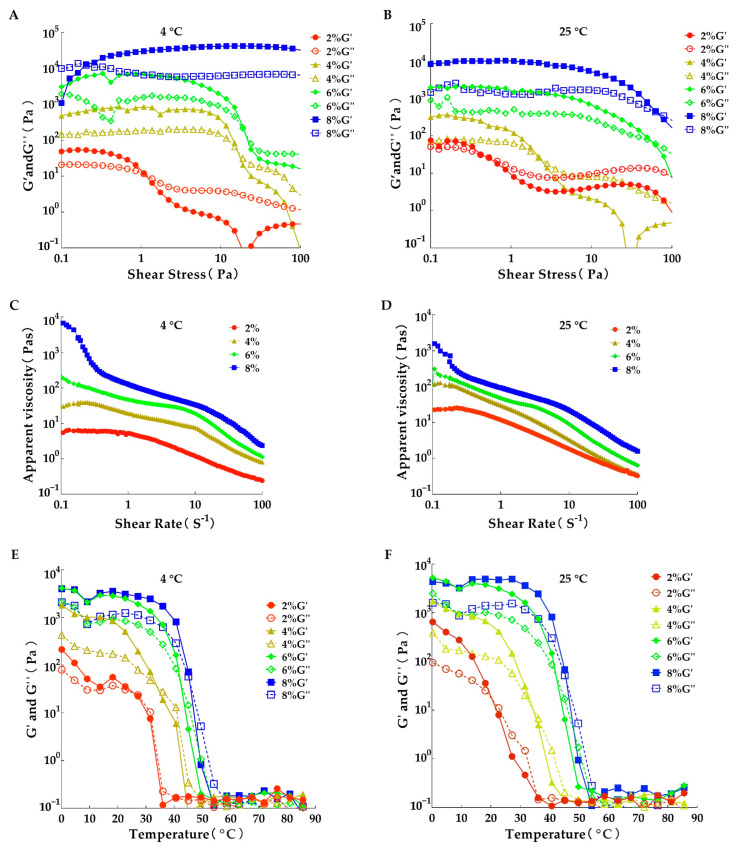
The effects of GMS concentration and storage temperature on the rheological properties of oleogels containing 15 μL/mL EOs. (**A**,**B**): stress sweep test; (**C**,**D**): shear rate sweep test; and (**E**,**F**): temperature sweep test during the cooling process. (**A**,**C**,**E**): stored at 4 °C for 24 h before testing; (**B**,**D**,**F**): stored at 25 °C for 24 h before testing.

Figure 4A–D illustrates the dynamic moduli and viscosity measurements of oleogels at storage temperatures of 4 and 25 °C, with shear rates ranging from 0.1 to 100 s^−1^ for each sample. The frequency dependence of G′ and G″ can be categorized into four types: dilute solutions, entanglement networks, weak gels, and strong gels. In the dilute solution phase, G″ exceeds G′ across the entire frequency range. The entanglement network phase is characterized by the intersection of the G′ and G″ curves within the tested frequency range. In the weak gel phase, G′ is greater than G″, with both moduli nearly parallel. Conversely, the strong gel phase also shows G′ exceeding G″, but with the slope of the G′ curve approaching zero and G″ reaching a minimum value at an intermediate frequency.

As shown in Figure 4A,B, at a GMS concentration of 8% and a storage temperature of 4 °C, G′ consistently exceeds G″, with both moduli nearly parallel, classifying it as a weak gel. All other oleogels exhibited intersections within the frequency range, transitioning from G′ exceeding G″ to G″ exceeding G′, indicating an entanglement network phase. Moreover, as the GMS concentration increased, the frequency resistance of the oleogels progressively increased. These findings suggest that the oleogels exhibit high resistance to deformation under stress, maintaining their solid structure.

Figure 4C,D reveals that the G′ and G″ values of samples containing 2% monostearate were significantly lower than those of other samples, indicating that GMS crystals at low concentrations disintegrated rapidly as the temperature increased. Based on the G′ and G″ values, it is evident that all oleogels exhibit elastic gel-like behavior and behave as shear-thinning pseudoplastic fluids, as G′ values consistently exceed their corresponding G″ values.

As shown in Figure 4E,F, the samples exhibited similar rheological behaviors. The shear-temperature curves of the oleogels can be divided into three distinct phases. Phase I involves heating from 0 °C to approximately 30 °C, during which G′ and G″ values decrease steadily. In Phase II, from 30 °C to 60 °C, G′ and G″ values decrease sharply, likely due to the increased phase behavior of Eos from thirteen spices, corn oil, and GMS crystals, as intermolecular attraction diminishes with rising temperature. In Phase III (60–90 °C), the oleogels completely melt. The temperature tolerance of the samples increased with higher GMS concentrations. A rapid decrease in G′ and G″ was observed at approximately 40 °C for oleogels with varying concentrations, consistent with the DSC results (Table 3—T_2_, T_4_). This also confirmed that the first peak in the DSC analysis corresponded to the transition of the hydrogel network to a liquid state.

### 3.6. X-Ray Diffraction Analysis

The process of oleogel formation can be divided into three phases: crystal nucleation, crystal branching, and crystal growth [33]. XRD was employed to examine the spacing, enabling precise characterization of cell parameters and specific crystal types. The plasticity of fats is primarily determined by their internal crystal packing mode and structure. At the molecular level, the fat crystal network is composed of various sub-crystalline structures formed through the arrangement and accumulation of vinyl groups within the fatty acid alkyl chains, a phenomenon referred to as homopolycrystallinity [34].

**Figure 5:**
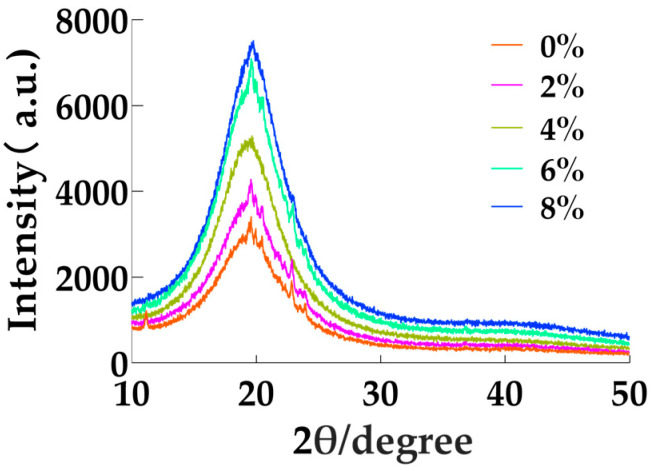
X-ray diffraction spectra of various concentrations of GMS oleogels containing 15 μL/mL of EOs.

There are three common types of fat crystals: α, β, and β’. Among these, α crystals are hexagonal, exhibit poor stability, and have low melting points. No diffraction peaks corresponding to α crystals were detected in the essential oil oleogels (Figure 5). β’ crystals, on the other hand, feature an orthogonal vertical subcellular stacking structure with uniform, fine needle-like crystals and a moderate melting point. These characteristics make β’ crystals ideal for the production of plastisols, as they provide a smooth texture and high spreadability [35].

According to the literature, the short distance peak at 0.42 nm is characteristic of α-type crystals, while strong diffraction peaks at 0.38 nm and 0.42 nm are indicative of β-type crystals. Additionally, the strong diffraction peak at 0.47 nm represents the characteristic peak of β’-type crystals [36]. As shown in Figure 5, the GMS content did not alter the crystal structure of the system. The diffraction patterns of oleogels with five different GMS concentration gradients were similar, indicating the presence of both β and β’ crystals. Moreover, the intensities of these peaks increased with higher GMS concentration, suggesting an increase in the number of crystals.

β’-type crystals are particularly noteworthy for their ability to encapsulate air and retain large quantities of liquid fat, forming a fine three-dimensional network. This makes them a promising candidate for use as plastic fats [37].

## 4. Conclusions

In this study, oleogels were successfully prepared using corn oil and EOs from thirteen spices as base oils. Oleogels were formed at various storage temperatures with GMS concentrations of 2% or higher. The GMS content significantly influenced the texture, OBC, as well as the DSC and XRD properties of the oleogel system. As the GMS concentration increased from 2% to 4%, the hardness of the oleogels at 4° C increased from 35.83 N to 128.97 N. Higher GMS content also enhanced the OBC and thermal stability of the oleogels.

XRD analysis revealed that the GMS content had minimal impact on the crystal morphology of the oleogels, with both β- and β’-type crystals present. When the GMS concentration reached 8%, the crystallinity of the oleogels increased, and their thermodynamic properties became more stable. Additionally, as the GMS content increased, the crystal density of the oleogels also rose, and the crystal distribution became more uniform, resulting in the formation of a dense network structure.

This study investigated oleogels based on EOs from thirteen spices, providing theoretical and technical support for their structural modification and regulation. This opens possibilities for the application of these EO-based oleogels in the meat industry [38]. The incorporation of spice EOs could not only replace unhealthy saturated fats but also enhance the flavor profile and potentially provide antioxidant or antimicrobial benefits to meat products. However, before widespread application, further research is needed to assess the gastrointestinal digestibility and long-term bioactivity stability of these EO-based oleogels from thirteen spices, particularly at varying concentrations of GMS. Investigating the interaction of these oleogels with meat matrices during processing and storage is also critical to ensure optimal product quality and consumer acceptance. With continued research and development, these oleogels have the potential to enhance food diversity, improve public health, and contribute to the sustainable evolution of the meat industry.

## Figures and Tables

**Table 1:** Color values of various concentrations of EOs oleogels (all loaded with 8% GMS).

Temperature (°C)	Concentration (μL/mL)	L*	a*	b*
4	0	11.07 ± 0.70 ^b^	−0.53 ± 0.15 ^d^	3.57 ± 0.64 ^b^
	5	12.73 ± 2.20 ^ab^	0.87 ± 0.21 ^c^	5.23 ± 0.42 ^a^
	10	12.87 ± 0.12 ^ab^	1.17 ± 0.06 ^bc^	5.43 ± 0.23 ^a^
	15	12.97 ± 0.68 ^ab^	1.33 ± 0.21 ^b^	5.47 ± 0.15 ^a^
	20	13.33 ± 0.71 ^ab^	1.50 ± 0.30 ^b^	5.7 ± 0.10 ^a^
	25	14.23 ± 1.24 ^a^	2.10 ± 0.01 ^a^	5.90 ± 0.44 ^a^
25	0	13.17 ± 0.59 ^a^	−0.53 ± 0.06 ^e^	3.5 ± 0.20 ^b^
	5	13.37 ± 0.94 ^a^	0.47 ± 0.15 ^d^	5.97 ± 0.50 ^a^
	10	13.57 ± 0.40 ^a^	0.97 ± 0.15 c	6.00 ± 0.40 ^a^
	15	13.63 ± 0.90 ^a^	1.40 ± 0.17 ^b^	6.57 ± 0.21 ^a^
	20	14.10 ± 0.10 ^a^	1.70 ± 0.10 ^b^	6.63 ± 0.31 ^a^
	25	14.73 ± 1.00 ^a^	2.20 ± 0.20 ^a^	6.67 ± 0.38 ^a^

Note: different letters within the same column are significantly different (n = 3).

**Table 2:** Effects of GMS concentration and storage temperature on the texture properties of oleogels containing 15 μL/mL EOs.

Temperature (°C)	Concentration (*w*/*w*,%)	Hardness (N)	Brittleness (N)	Viscosity (N.ges)	Chewiness (N)
4	2	35.83 ± 7.21 ^a^	31.9 ± 4.39 ^a^	15.51 ± 3.65 ^a^	15.40 ± 3.74 ^a^
	4	128.97 ± 27.13 ^a^	125.70 ± 25.61 ^a^	33.01 ± 12.36 ^a^	32.37 ± 12.05 ^ab^
	6	323.77 ± 33.03 ^b^	325.67 ± 21.41 ^b^	64.12 ± 10.19 ^ab^	63.84 ± 10.09 ^ab^
	8	437.66 ± 79.50 ^c^	427.20 ± 75.88 ^b^	110.56 ± 39.77 ^b^	95.04 ± 54.52 ^b^
25	2	20.32 ± 1.05 ^a^	18.05 ± 3.09 ^a^	11.86 ± 1.96 ^a^	11.75 ± 2.01 ^a^
	4	62.36 ± 2.92 ^b^	49.06 ± 10.75 ^a^	29.29 ± 2.80 ^b^	29.11 ± 2.89 ^b^
	6	211.79 ± 31.90 ^c^	197.40 ± 28.63 ^b^	45.96 ± 3.04 ^c^	45.79 ± 2.88 ^c^
	8	332.00 ± 22.07 ^d^	262.24 ± 112.88 ^c^	99.48 ± 9.60 ^d^	94.50 ± 13.01 ^d^

Note: different letters within the same column are significantly different (n = 3).

**Table 3:** Phase transition temperatures of various concentrations of GMS oleogels containing 15 μL/mL EOs.

GMS Concentration (*w*/*w*,%)	T_1_ (°C)	T_2_ (°C)	T_3_ (°C)	T_4_ (°C)	T_5_ (°C)
2	13.48 ± 1.29 ^a^	14.60 ± 1.36 ^a^	30.76 ± 3.27 ^b^	38.58 ± 7.68 ^b^	--
4	13.50 ± 1.07 ^a^	14.73 ± 1.09 ^a^	39.26 ± 1.59 ^b^	44.06 ± 8.12 ^b^	--
6	13.72 ± 0.93 ^a^	14.95 ± 0.99 ^a^	45.91 ± 2.45 ^b^	48.99 ± 7.14 ^b^	62.85 ± 3.48 ^c^
8	13.75 ± 0.92 ^a^	14.97 ± 0.97 ^a^	48.69 ± 1.55 ^b^	51.01 ± 6.99 ^b^	63.96 ± 2.96 ^c^

Note: different letters within the same column are significantly different (n = 3). T1, lower peak temperature; T2, lower peak temperature; T3, higher peak temperature; T4, higher peak temperature; T5, higher peak temperature.

## Data Availability

The original contributions presented in the study are included in the article; further inquiries can be directed to the corresponding author.

## Supplementary Materials

The following supporting information can be downloaded at https://www.mdpi.com/article/10.3390/foods14030542/s1, Table S1. Composition of Thirteen-Spice Essential Oil by GC-MS.
